# A light-weight Graph Neural Network for the prediction of ^31^P Nuclear Magnetic Resonance signals

**DOI:** 10.1186/s13321-026-01178-6

**Published:** 2026-03-17

**Authors:** Dimitri Domnjuk, Jana de Wiljes, Robert Geitner

**Affiliations:** 1 Institute of Chemistry and Bioengineering, Group of Physical Chemistry/Catalysis, Technische Universität Ilmenau, Weimarer Str. 32, 98693 Ilmenau, Germany; 2 Institute of Mathematics, Group of Mathematics of Data Science, Technische Universität Ilmenau, Weimarer Str. 25, 98693 Ilmenau, Germany; 3 School of Engineering Sciences, Department of Computational Engineering, LUT University, Yliopistonkatu 34, 53850 Lappeenranta, Finland

**Keywords:** NMR, Graph Neural Network, Phosphorous, Prediction, Transfer learning, HOSE

## Abstract

Graph Neural Networks (GNNs) are powerful tools for predicting chemical shifts in Nuclear Magnetic Resonance (NMR) spectroscopy. In this paper, we improve the state-of-the-art mean absolute error (MAE) on the Ilm-NMR-P31 dataset for the prediction of ^31^P shifts from 11.4 ppm to 8.88 ppm by proposing a lightweight GNN which is based on the Metalayer-Framework. Furthermore, we analyze the performance of our model depending on the size of the training dataset and compare our model with different state-of-the-art models. Finally, we demonstrate how the GNN’s predictions can be interpreted and visualized with respect to the underlying molecular structures. Using GNNExplainer, we analyze the best- and worst-predicted molecules and perform feature ablations to assess the model’s reliance on specific input features. Furthermore, we validate the model’s physical plausibility by extracting learned substituent effects: the GNN autonomously rediscovers established empirical rules, accurately reproducing the $\beta$-deshielding and $\gamma$-shielding increments of neutral phosphines. Finally, we show that the SDGNN outperforms standard vacuum DFT calculations and HOSE code baselines.

**Scientific Contribution:** The proposed lightweight GNN based on the Metalayer framework improves the state-of-the-art ^31^P NMR shift prediction. By systematically varying the training-set size and benchmarking against multiple state-of-the-art models, we provide a standardized performance comparison that was previously lacking for ^31^P NMR shift prediction. Using GNNExplainer and targeted feature ablations, we relate the model’s predictions to specific molecular substructures and input features. By quantitatively verifying that the model learns fundamental physical trends like substituent increments and identifying specific error sources, we provide a level of chemical interpretability and validation that goes beyond prior black-box approaches.

**Supplementary Information:**

The online version contains supplementary material available at 10.1186/s13321-026-01178-6.

## Introduction

Phosphorus-containing compounds play a key role across many areas of chemistry and biochemistry. Additionally, phosphorus is widely used in industrial applications, such as catalysis [1, 2] and pharmaceutical synthesis [3]. Understanding how phosphorus behaves in different molecular environments requires insights into its structure and electronic properties [4]. One widely used tool for this is nuclear magnetic resonance (NMR) spectroscopy, in which the ^31^P chemical shift provides valuable information how phosphorus atoms interact with their surroundings [5].

^31^P is a commonly studied nucleus in NMR spectroscopy as this isotope has a natural abundance of 100% and a gyromagnetic ratio of 17.235 MHz T^-1^ [6], which makes ^31^P one of the more sensitive NMR-active nuclei. Nevertheless, phosphorus-containing compounds are rare in comparison to hydrogen- and carbon-containing molecules. Therefore, ^1^H and ^13^C NMR data is more readily available, and consequently the prediction of ^1^H and ^13^C NMR signals is also better understood [7].

Accurate prediction of chemical shifts is becoming increasingly important as computer-assisted structure elucidation (CASE) systems become more widespread and automated data analysis protocols gain popularity, supporting the growing potential of robot-assisted high-throughput experimentation [7, 8]. For NMR shift prediction the best performing systems are currently graph neural networks (GNNs) [7]. A GNN is a specialized neural network designed to handle graph-structured data [9], making them particularly well-suited for chemical applications, as they naturally represent molecules as graphs with atoms being nodes and bonds being edges. Kuhn et al. designed a convolutional GNN and reached Mean Absolute Errors (MAEs) of 0.28 and 1.43 ppm for ^1^H and ^13^C [10] while Guan et al. studied 3D GNNs and reached a MAE of 0.10 and 1.26 ppm for ^1^H and ^13^C nuclei, respectively [11].

However, the interpretation of molecular structure in NMR-related tasks remains a challenge even for advanced artificial intelligence systems. For instance, a recent benchmark by Jablonka et al. found that leading Large Language Models struggled significantly with predicting the number of signals in an NMR spectrum based solely on Simplified Molecular Input Line Entry System (SMILES) strings, achieving low accuracy due to difficulties in reasoning about molecular symmetry and topology [12]. This underscores the need for models that can effectively capture and reason about detailed structural information, a prerequisite for accurate chemical shift prediction.

One major limitation of GNNs in the context of NMR shift prediction is the reliance on a relatively small amount of experimental data. Even for ^1^H and ^13^C the available databases list only a few hundred thousand individual atomic shift values, which is a modest number compared to neural networks are usually trained on much larger datasets [13]. Common open-access NMR databases include nmshiftdb2 [14] and Spectral Database for Organic Compounds (SDBS) [15]. This issue becomes even more pronounced for heteronuclear NMR spectroscopy. For ^31^P only 14,250 data points are openly available in the Ilm-NMR-P31 dataset [16] while for ^19^F nmrshiftdb2 lists only 2,076.

Besides the tedious process of synthesizing and measuring new molecules to expand the data foundation, models that are specifically designed to handle small datasets or leverage large auxiliary datasets through innovative training paradigms are of particular interest. For instance, Xu et al. recently demonstrated with their NMRNet framework that pretraining SE(3) (Special Euclidean Group in 3-dimensions) Transformer models on vast structural databases, followed by fine-tuning on NMR-specific data, significantly enhances prediction accuracy across various nuclei, setting a new standard for leveraging diverse data sources [17]. Recently Kuhn et al. tackled this data sparsity challenge by designing a message-passing graph network, which in accordance with the original report we name the "2023 Model". The 2023 Model was able to outperform a convolutional GNN when fewer than 5,000 ^13^C training data points were available [18].

To address the limitation of sparse data, we introduce the Small Data Graph Neural Network (SDGNN) model. The SDGNN is a parameter-efficient architecture based on the MetaLayer framework [19], designed for rapid inference and strong performance on limited training data. It was benchmarked against three established models: the Nuclear Magnetic Resonance Message-Passing Neural Network “nmrMPNN” [20], a reimplemented version of the “2023 Model by Rull, Kuhn, and Fischer [18], which was adapted from its original local prediction task to the present graph-level prediction setting, as well as against Hierarchically Ordered Spherical Description of Environment (HOSE) code-based prediction [21]. To the best of our knowledge, the SDGNN achieves the most accurate ^31^P shift predictions reported to date, while simultaneously exhibiting a dramatically reduced parameter count, which accelerates training and lowers energy consumption. We will compare the performance of our SDGNN to existing approaches like Hierarchically Ordered Spherical Environment (HOSE) codes [21], traditional GNNs and the 2023 Model applied to ^31^P shift prediction.

## Methods

### Data processing and feature selection

As our data source, we utilized the Ilm-NMR-31P dataset [16], which provides 14,250 experimentally determined ^31^P NMR shifts containing 13,730 distinct molecules from 3,648 references. We retained these multiple entries in our training process, as they represent legitimate experimental variance, thereby encouraging the model to learn robust representations of the chemical shift. Molecular structures were derived from the Ilm-NMR-31P dataset, using SMILES strings, which were converted into molecular graphs using the RDKit library [22]. All our molecular graphs are 2D graphs and no geometrical features were used.

Regarding the Signal-to-noise ratio of the underlying spectral data, the dataset relies on curated values from established handbooks, assuming a baseline quality ensured by the original peer-review processes. However, the dataset exhibits label noise due to the absence of experimental solvent, concentration, temperature information and field strength, which was generally not available in the source material [16]. Consequently, our model learns to predict chemical shifts generalized across these unknown experimental variations, effectively treating solvent effects as aleatoric uncertainty.

To ensure high structural fidelity, the original dataset curators prioritized manual verification over automated extraction. While optical character recognition (OCR) was used for data mining, molecular structures were largely hand-drawn and manually corrected to resolve OCR inconsistencies. A manual validation of a random subset of 1% of the database, performed by Hack et al. showed no structural or referencing errors [16].

**Fig. 1:**
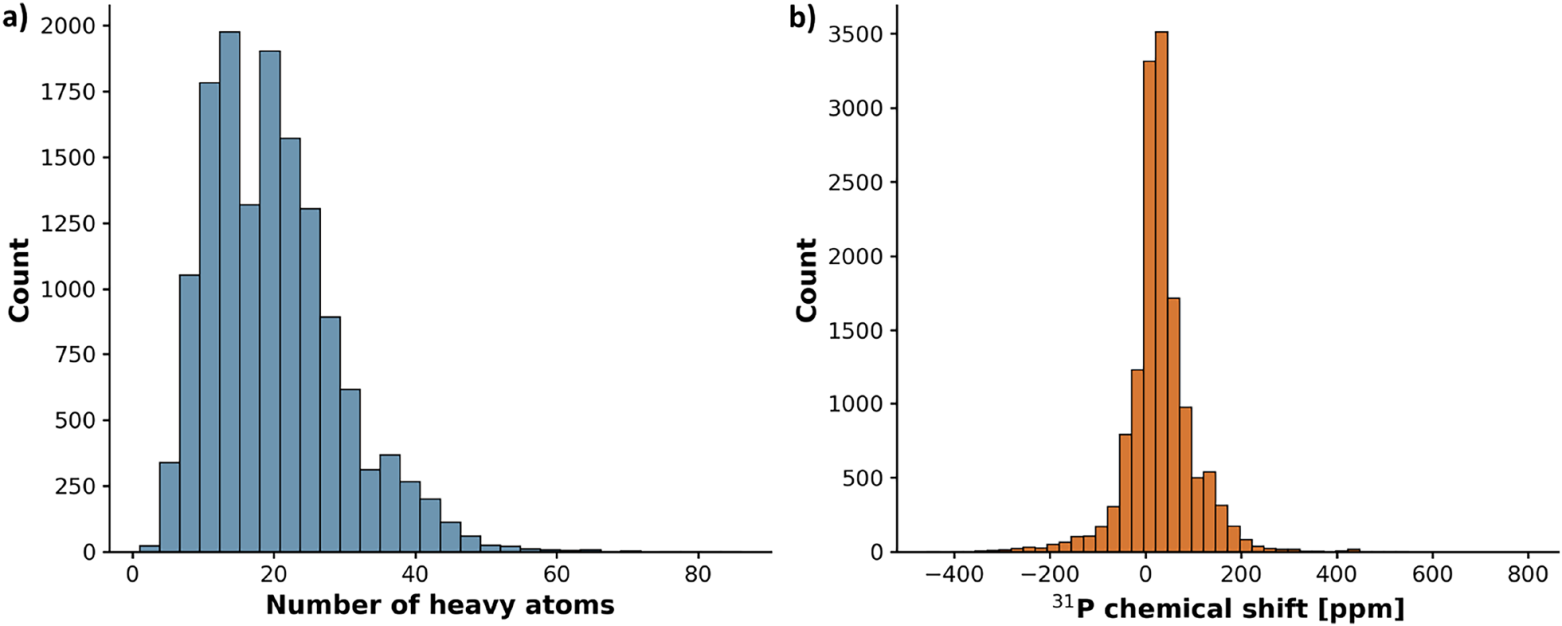
Distribution of molecular sizes measured by the number of non-hydrogen atoms **a** as well as the distribution of experimental $^{31}$P chemical shifts **b** covering a broad spectral range typical of organic phosphorus chemistry

An analysis of the dataset composition reveals a high chemical diversity suitable for training robust machine learning models. The experimental chemical shifts cover a broad spectral width ranging from $-457$ to $+800$ ppm, with a mean of 30.05 ppm. Structurally, the dataset is dominated by organophosphorus compounds, specifically phosphonates and tertiary phosphines, as well as phosphates and phosphonium salts. The molecules vary in size with a median of 13 carbon atoms per molecule [16]. The molecules are relatively compact, with a mean of 19.92 and a median of 18.0 heavy atoms per graph, and a maximum size of 86 nodes. In terms of node features, the dataset includes 35 different element types. Apart from the core atoms Phosphorus which is present in all graphs, Carbon with $\approx$ 196k nodes, Oxygen with $\approx$ 33k nodes, and Nitrogen with $\approx$ 15k nodes, the dataset contains significant numbers of halogens and other heteroatoms, with Fluorine, Chlorine, Sulfur, Silicon, and Bromine being the most frequent (Fig. 1).

To validate the generality of the dataset, we analyzed its structural diversity using Bemis-Murcko scaffolds [23] and Morgan fingerprints using the Extended-Connectivity Fingerprint (ECFP4) algorithm [24]. The dataset contains 3,755 unique molecular scaffolds among the 14,062 valid compounds, resulting in a scaffold diversity score of 0.264. This reflects a substantial degree of topological variety, indicating that the dataset extends beyond simple substituent modifications of a small number of core structures.

**Fig. 2:**
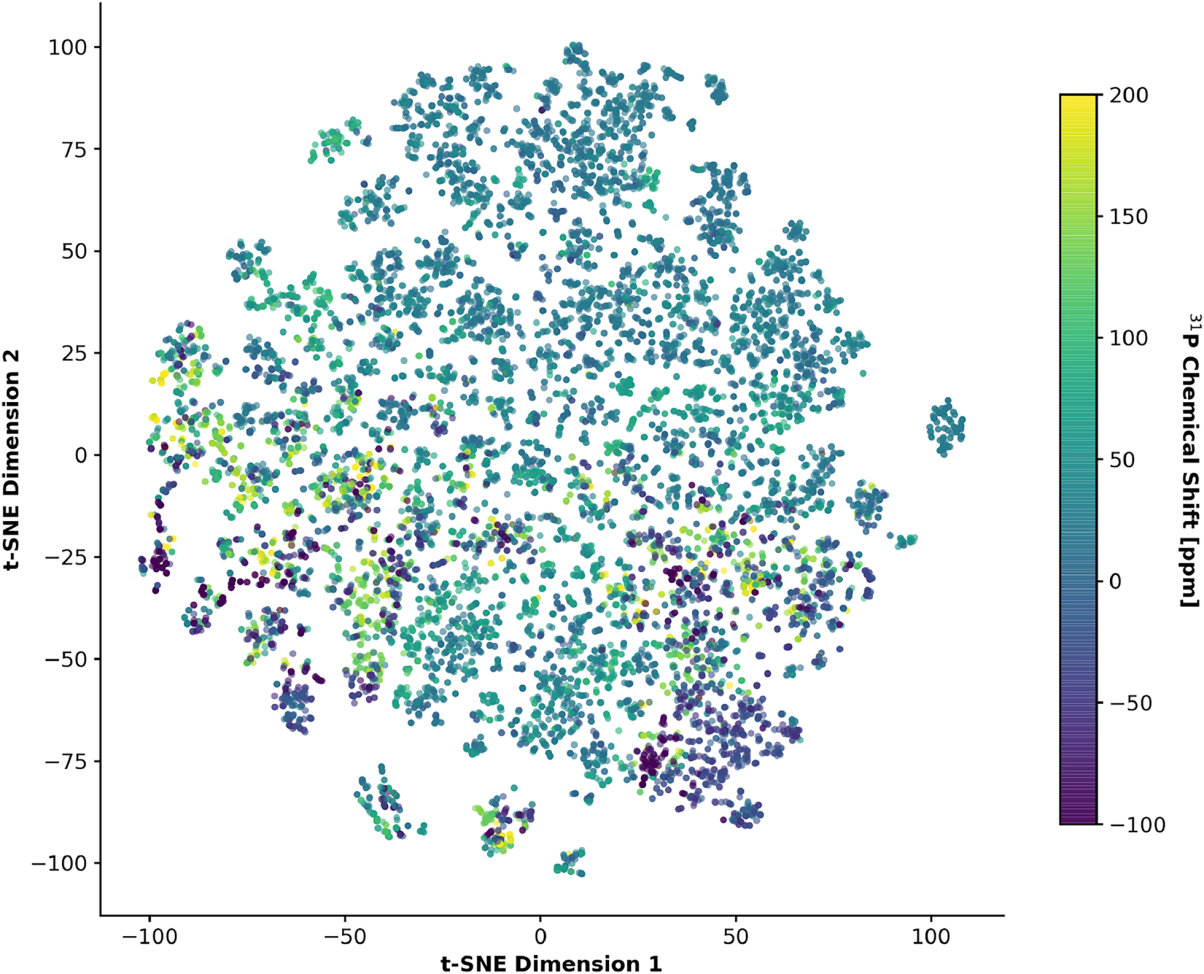
A t-SNE projection of 14,062 molecules based on 2048-bit Morgan fingerprints with a radius of 2. Each point represents a molecule, colored by its experimental $^{31}$P chemical shift. Local clustering of similar colors suggests underlying structure–property relationships, while the overall spread of the embedding is consistent with a chemically diverse training set

In addition, we explored the distribution of molecular structures using t-Distributed Stochastic Neighbor Embedding (t-SNE) applied to the fingerprint representations. The resulting embedding exhibits a structured chemical space with localized clusters that are consistent with distinct molecular families. Coloring the projection by experimental $^{31}$P chemical shifts reveals non-random spatial patterns, suggesting underlying structure–property relationships across the dataset. Taken together, these observations are consistent with a chemically diverse dataset that provides a suitable basis for learning structure-dependent $^{31}$P chemical shift trends (Fig. 2).

Formal definitions for all three GNNs (SDGNN, 2023 Model and nmrMPNN) can be found in the Supporting Information.

We derived 29 node features, nine edge features, and nine global features from the molecular graphs by quantifying the contributions of node, edge, and global features using a noise injection based feature ablation. More specifically, for each feature dimension in the node, edge and global feature vectors, we generated perturbed versions of the test data by replacing the scaled original feature values with random noise sampled from a normal distribution $\mathcal {N}(0, 1)$. By comparing the MAE of the perturbed SDGNN model to that of the baseline with the unmodified features, we calculated the degradation in performance, $\Delta \text {MAE}$, as an estimate of the importance of the ablated feature. A larger increase in MAE thus indicates a higher dependency of the model on that particular feature.

Node features characterize individual atoms within the molecular graph. These features capture a wide range of atomic properties, such as atomic number, valence, hybridization, and various chemical descriptors obtained from both RDKit [22] and Mendeleev [25]. This comprehensive feature set enables the model to understand the chemical environment of each atom in detail.

Edge features represent the bond properties connecting the atoms. They encode information about the bond order, whether the bond is part of a ring, its conjugation status, aromaticity, and stereochemistry, as well as additional properties that quantify the differences and interactions between the connected atoms.

Global features summarize the overall properties of the molecule. These include counts and ratios of specific atom types, global measures such as the Topological Polar Surface Area (TPSA), molar refractivity, and various aggregated statistics that reflect the molecular composition and geometry. For a complete list of all features see Tables 1, 2 and 3.

Node features, edge features, and global features were separately scaled using the StandardScaler from sklearn [26], which standardizes features by subtracting the mean and scaling to unit variance.

**Table 1:** Node features used in molecular graphs

Feature name	Description
Atomic number	The atomic number of the atom
Degree	Number of adjacent atoms [22]
Atomic radius	Atomic radius from Mendeleev’s data [25]
Atomic volume	Atomic volume from Mendeleev’s data [25]
Formal charge (one-hot)	One-hot encoding of the formal charge [22]
Covalent radius	Covalent radius from Mendeleev’s data [25]
Van der Waals radius	Van der Waals radius from Mendeleev’s data [25]
Dipole polarizability	Dipole polarizability from Mendeleev’s data [25]
Electron affinity	Electron affinity from Mendeleev’s data [25]
Electrophilicity	Electrophilicity index [25]
Enthalpy/Pauling electronegativity	Pauling electronegativity [25]
Number of electrons	Total number of electrons [25]
Number of neutrons	Total number of neutrons [25]
Chiral tag	Chirality indicator (encoded as integer) [22]
In ring	Binary flag indicating if the atom is in a ring [22]
Aromatic	Binary flag for aromaticity [22]
Hybridization (one-hot)	One-hot encoding of the hybridization state [22]
Mass	Atomic mass [22]
Explicit valence	Explicit valence of the atom [22]
Total valence	Total valence of the atom [22]
Ring size (atom)	Size of the smallest ring the atom is part of [22]
Gasteiger charge	Partial charge computed by the Gasteiger method [22]
Number of rings	Count of rings in which the atom is involved [22]
Is phosphorus	Binary flag: 1 if the atom is phosphorus, else 0
Aromatic bond count	Number of aromatic bonds the atom is involved in [22]
Sum of bond orders	Total bond order sum for bonds involving the atom [22]
Heavy neighbor count	Count of neighboring atoms (non-hydrogen) [22]
Non-carbon neighbor count	Count of neighbors that are not carbon [22]
Charge difference	Gasteiger charge minus the formal charge [22]

**Table 2:** Edge features used in molecular graphs

Feature name	Description
BondType	Bond order (e.g., single, double, triple) [22]
IsInRing	Boolean indicating if the bond is part of a ring [22]
Conjugated	Boolean indicating if the bond is conjugated [22]
Is aromatic	Boolean indicating if the bond is aromatic [22]
RingSize	Size of the smallest ring containing the bond [22]
Stereo	Stereochemistry of the bond [22]
Is rotatable	1 if the bond is a single bond and not in a ring, else 0 [22]
Abs. Degree difference	Absolute difference of the degrees of the two bonded atoms [22]
Sum of degrees	Sum of the degrees of the two bonded atoms [22]

**Table 3:** Global features used in molecular graphs

**Feature name**	**Description**
Number of nitrogen	Total number of nitrogen atoms in the molecule
Ratio of phosphorus	Fraction of phosphorus atoms relative to total atoms
Formal charge	Overall formal charge of the molecule [22]
TPSA	Topological Polar Surface Area [22]
Molar refractivity	Molar refractivity of the molecule [22]
Number of aromatic rings	Count of aromatic rings in the molecule [22]
Ratio of aromatic atoms	Fraction of atoms that are aromatic [22]
Average mass	Average atomic mass of the atoms in the molecule [22]
Aromatic count	Total number of aromatic atoms in the molecule [22]

### Model training and evaluation

To assess the performance of our models, three key metrics were considered. The Mean Absolute Error (MAE) is defined as:

1 $$\begin{aligned} \text {MAE} = \frac{1}{N} \sum _{i=1}^{N} | y_i - \hat{y}_i | \end{aligned}$$

where $y_i$ represents the true chemical shift value and $\hat{y}_i$ denotes the predicted value. The MAE provides an intuitive measure of the average absolute deviation between predicted and actual shifts. The Root Mean Squared Error (RMSE) is computed as:

2 $$\begin{aligned} \text {RMSE} = \sqrt{\frac{1}{N} \sum _{i=1}^{N} (y_i - \hat{y}_i)^2} \end{aligned}$$

Besides these error measures the number of trainable parameters in millions (M) for each model was used as an indicator of model complexity. The MAE was used as the loss function during training. Since we use k-fold validation, for each fold the MAE was computed on the validation set. Final performance was reported as the average metric loss across all three folds. The hyperparameter optimization and model training were conducted on the Massive Parallel Compute Cluster (HPC) at Technische Universität Ilmenau. Specifically, tasks were executed on compute nodes within the GPU partition, which are based on x86_64 architecture and primarily feature AMD EPYC series processors, with each job task allocated four CPU cores. The GPU nodes are equipped with NVIDIA A100 GPUs and between 512 and 1024 GiB of RAM, running CentOS Linux 7 (Core) as the operating system. The experiments were performed using Python 3.12.10, together with key scientific and cheminformatics libraries including pandas 2.2.3 [27], NumPy 2.2.6 [28], RDKit 2024.09.5 [22] for molecular structure processing, PyTorch 2.5.1+cu121 [29] for deep learning, PyTorch Geometric 2.6.1 [30, 31] for graph-based neural networks, torch-scatter 2.1.2+pt25cu121 [29] for efficient scatter operations, and Mendeleev 0.20.1 [25] for chemical element data. Data visualization and model evaluation were supported by matplotlib 3.10.3 [32] and scikit-learn 1.6.1 [26]. Further data visualization was done using R 4.4.1 [33] as well as its packages tidyverse 2.0.0 [34] and scales 1.3.0 [35]. To optimize the performance of the models, a systematic hyperparameter search using grid search in combination with 3-fold cross-validation and early stopping was performed.

While the training and hyperparamter optimization were performed on random splits, all test runs were evaluated using chemically informed data splits to prevent optimistic bias. In addition to group-based splits that ensure identical molecular structures, via canonical SMILES, we employed Bemis-Murcko scaffold splits [36] to prevent closely related molecular features from appearing across training, validation, and test sets. For each setting, performance is reported as the mean ± standard deviation for the different dataset sizes $k \in \{100; \ 500; \ 1,000; \ 2,500; \ 5,000; \ 14,062\}$ each of which was calculated via ten independent folds. We always use 80% of the graphs as training data, 10% for validation and 10% for testing. In addition to the models evaluated here, previous work on the same Ilm-NMR-P31 dataset investigated a range of classical non-graph machine learning baselines, including fingerprint-based random forests and gradient boosting models. These approaches were consistently found to underperform both HOSE code-based prediction and GNNs, and are therefore not reimplemented in this work [16]. Therefore we compared the GNN models with a well established, non-graph based Baseline: HOSE codes, which were trained following the same chemical informed splits as the GNNs.

The hyperparameters were optimized using a grid search, including the learning rate (lr; 0.01, 0.001, 0.0001), weight decay (wd; 0, 0.0001), batch size (bs; 16, 32, 48), and the hidden layer dimension (hd; 32, 64, 128, 256). Early stopping was controlled via the patience parameter (pat), set to either 40 or 200 epochs without improvement, while the total number of training epochs (ep) was fixed at 500. Additionally, the number of message passing rounds (mp) in the graph neural networks was optimized over 1, 2, and 3 rounds. In contrast to the SDGNN, which was intentionally designed to perform a single message-passing round, the number of message-passing iterations was treated as a tunable hyperparameter for the other GNN architectures. All possible combinations of the hyperparameters were generated using grid search. For each combination, 3-fold cross-validation was performed on the training data. During each fold, the model was trained and validated, and the validation loss was recorded. The training process for each fold incorporated early stopping; if the validation loss did not improve for a specified number of epochs (as determined by the patience parameter), training was halted early. The best-performing model (i.e., the one with the lowest validation MAE) was saved for each fold. After cross-validation, the hyperparameter configuration with the lowest average validation loss across all folds was selected as the optimal setting. The results can be seen in Tables 4, 5 and, 6. Finally, each model including the HOSE code-based model was re-initialized with the best hyperparameters from the grid search and evaluated on the test set using the MAE and RMSE on ten separate folds using chemical informed splits described above.

**Table 4:** Best hyperparameter combination and MAE per model and subset size averaged out on 3-folds for the SDGNN model

Model	$\textbf{k}$	MAE (ppm)	lr	wd	bs	hd	pat	ep
SDGNN	100	24.26	0.01	0.0001	16	128	200	500
	500	19.87	0.01	0	16	32	200	500
	1000	20.38	0.01	0	32	128	200	500
	2500	16.45	0.01	0	48	32	200	500
	5000	14.39	0.01	0	48	64	200	500
	14062	9.51	0.001	0.0001	48	256	200	500

**Table 5:** Best hyperparameter combination and MAE per model and subset size averaged out on 3-folds for the 2023 model

Model	$\textbf{k}$	MAE (ppm)	lr	wd	bs	hd	pat	ep	mp
2023 Model	100	22.22	0.01	0.0	16	32	200	500	1
	500	20.28	0.001	0.0	16	64	200	500	1
	1000	20.44	0.001	0.0	16	64	200	500	2
	2500	17.93	0.001	0.0	16	128	40	500	1
	5000	14.68	0.001	0.0	16	128	200	500	1
	14062	9.87	0.0001	0.0	48	256	200	500	1

**Table 6:** Best hyperparameter combination and MAE per model and subset size averaged out on 3-folds for the nmrMPNN model

Model	$\textbf{k}$	MAE (ppm)	lr	wd	bs	hd	pat	ep	mp
nmrMPNN	100	23.73	0.0010	0.0001	48	64	200	500	1
	500	20.92	0.0001	0.0001	32	128	40	500	2
	1000	17.29	0.0001	0.0	16	64	40	500	3
	2500	16.82	0.0001	0.0	16	128	40	500	1
	5000	12.75	0.0001	0.0	16	256	200	500	2
	14062	9.62	0.0001	0.0001	16	256	200	500	3

### Model interpretation using the GNNExplainer

To interpret the decision-making process of the model, the GNNExplainer [37] was employed to analyze individual predictions of the nmrMPNN model obtained from the hyperparameter optimization. The model had been trained on 10,000 data points and achieved a MAE of 9.54 on the test set. GNNExplainer is an optimization-based approach that identifies a compact subgraph and a subset of node features most influential for a given prediction. For our analysis, the explainer was configured to learn two distinct soft masks. The edge mask was set to object, assigning a single importance scalar for each bond. Concurrently, the node mask was set to attributes, learning a distinct importance value for each individual feature of every atom. This configuration enabled a fine-grained assessment of which structural and atomic properties contributed most strongly to the model’s predictions. Mask optimization was performed over 200 epochs with a learning rate of 0.01, aiming to maximize the mutual information between the model’s prediction on the original graph and the masked subgraph, while simultaneously penalizing the mask sizes to encourage sparsity. The resulting atom-level importances which were summed across feature dimensions and bond importances were normalized and subsequently used to scale the node sizes and edge thicknesses in our visualizations.

### Chemical validation metrics

Beyond raw predictive performance, it is crucial to verify whether the SDGNN captures fundamental chemical principles or merely memorizes structures. For this, we performed an error analysis of the best models predictions across chemical subclasses and validated its ability to reproduce established physical rules for NMR shifts. First, we partitioned the test set into chemically meaningful subclasses using SMILES Arbitrary Target Specification (SMARTS) patterns implemented in RDKit [22]. Specifically, we distinguished four primary functional groups based on the local coordination environment of phosphorus: tertiary phosphines (P(III), R$_3$P), phosphine oxides (R$_3$P=O), phosphonates (RP(=O)(OR)$_2$), and phosphates ((OR)$_3$P=O). In addition, we flagged chemically challenging motifs, namely formally charged species (determined by rdkit) and silyl-substituted phosphorus centers (P-Si bonds), in order to isolate error contributions arising from missing counterion information and pronounced electronic effects.

Second, we quantified whether our SDGNN implicitly recovers classical substituent effects in $^{31}$P NMR spectroscopy. For this purpose, we selected homologous series of simple alkyl-substituted phosphorus compounds and fitted the model predictions $\delta _{\textrm{pred}}$ to the empirical linear increment model

3 $$\begin{aligned} \delta _{\textrm{pred}} = B + n_{\beta } A_{\beta } + n_{\gamma } A_{\gamma }, \end{aligned}$$

where *B* denotes the base chemical shift of the parent compound, $n_{\beta }$ and $n_{\gamma }$ are the numbers of carbon atoms in $\beta$ and $\gamma$ positions relative to phosphorus, and $A_{\beta }$ and $A_{\gamma }$ represent the corresponding substituent increments. The fitted coefficients $(B, A_{\beta }, A_{\gamma })_{\textrm{GNN}}$ obtained from SDGNN predictions were compared against experimentally reported reference shifts for representative phosphorus compound classes compiled by Berger et al. [38] This analysis enables a direct assessment of whether the network reproduces the well-known deshielding $\beta$-effect and shielding $\gamma$-effect, thereby providing a physically grounded validation of the learned representations.

## Results and discussion

A prominent trend in the HPO across all three GNN models is the direct relationship between dataset size and optimal model capacity (Tables 4, 5 and, 6). As the number of training samples *k* increases, the HPO consistently selects a larger hidden dimension indicating that larger models with higher capacity are required to capture the complex relationships present in larger datasets, while smaller models are preferred in data-limited regimes to prevent overfitting.

Concurrently, the chosen learning rate shows a clear inverse relationship with dataset size. For smaller *k*, a higher learning rate is favored, enabling faster convergence in a simpler loss landscape.

While the 2023 Model favors a single message-passing round, for the nmrMPNN the number of message passing rounds tends to increase with dataset size, scaling from one round at $k=100$ to three rounds at $k=1,000$ and $k=14,062$. This suggests that with more data, the model “sees a larger *k*-hop neighborhood around phosphorus, so it can capture 2-4-bond substituent effects, ring currents, and other medium-range influences on the ^31^P shifts. This is directly analogous to HOSE codes: *k* message passing rounds roughly corresponds to a HOSE level *k* (atoms within *k* bonds). The nmrMPNN architecture also concatenates intermediate states across rounds, effectively stacking HOSE levels into a learned multi-scale descriptor instead of a fixed fingerprint. Empirically, increasing the model’s receptive field yields performance gains only when the dataset is sufficiently large to prevent overfitting of the additional parameters (see Table 6). We identified a sweet spot at three message passing rounds, which aligns with the common practice of using HOSE code levels 3-4 for ^31^P NMR prediction [16].

**Table 7:** Prediction performance of graph-based GNNs and the HOSE code baseline on the Ilm-NMR-P31 dataset using molecule-disjoint cross-validation, grouped by canonical SMILES

Model	Metric	100	500	1000	2500	5000	14062
SDGNN	MAE (ppm)	25.45 ± 12.1	23.47 ± 6.3	20.20 ± 2.1	17.42 ± 1.6	14.59 ± 1.2	8.88 ± 0.4
	RMSE (ppm)	38.95 ± 19.2	45.58 ± 15.3	41.18 ± 10.5	36.27 ± 5.7	29.64 ± 4.0	22.41 ± 2.5
	Params (M)	0.10	0.01	0.10	0.01	0.03	0.36
2023 model	MAE (ppm)	26.77 ± 12.9	24.49 ± 4.3	21.02 ± 2.6	16.99 ± 1.6	13.95 ± 1.1	11.60 ± 0.6
	RMSE (ppm)	38.50 ± 20.9	43.77 ± 9.8	42.61 ± 10.8	36.76 ± 6.5	28.65 ± 4.1	27.04 ± 2.9
	Params (M)	0.02	0.06	0.06	0.23	0.23	0.88
nmrMPNN	MAE (ppm)	27.30 ± 12.5	29.87 ± 6.1	27.98 ± 4.8	18.04 ± 1.9	13.97 ± 1.3	9.87 ± 0.4
	RMSE (ppm)	41.01 ± 20.5	52.48 ± 14.9	55.72 ± 14.5	37.15 ± 5	30.37 ± 4.5	22.60 ± 1.7
	Params (M)	1.60	2.72	0.68	1.60	1.60	15.03
HOSE	MAE (ppm)	2.62 ± 2.3	13.52 ± 3.1	15.86 ± 3.7	12.26 ± 1.7	11.22 ± 0.9	10.22 ± 0.4
	RMSE (ppm)	3.10 ± 3.0	26.14 ± 9.4	35.12 ± 11.2	26.94 ± 5.9	25.73 ± 4.0	24.37 ± 2.1
	Failed (%)	80.0 ± 10.0	56.0 ± 5.7	45.5 ± 6.3	32.4 ± 1.8	23.6 ± 1.9	12.4 ± 0.7

Mean absolute error (MAE) and root mean squared error (RMSE) are reported as mean ± standard deviation across 10 independent folds

To properly interpret the reported prediction errors, it is important to relate them to the experimental tolerances inherent in ^31^P NMR spectroscopy. While instrumental precision allows for signal determination within 0.01 ppm [39], the chemical shift is highly sensitive to environmental factors. For instance, solvent effects alone can induce shifts of approximately 2.4 ppm for simple phosphates [40], while literature comparisons for identical compounds measured under varying conditions have reported deviations of up to 10 ppm [38]. Even highly controlled absolute shielding measurements in the gas phase, for example, for PH_3_ exhibit uncertainties of 0.63 ppm [41]. Since the Ilm-NMR-P31 dataset aggregates data from thousands of sources without consistently capturing solvent, concentration, or temperature metadata, these environmental variations constitute an irreducible (aleatoric) error. In this context, our model’s performance on the grouped split MAE of 8.88 ppm and specifically the median MAE of 1.80 ppm suggests that for the majority of molecules, the prediction error lies within the range of typical solvent variability (see Table 9). This suggests that for the majority of molecules, the SDGNN prediction error lies within the range of typical solvent and experimental condition variability of $1 - 3$ ppm. Larger deviations likely reflect the dataset’s intrinsic noise or strong intermolecular interactions not captured by the 2D molecular graph.

**Fig. 3:**
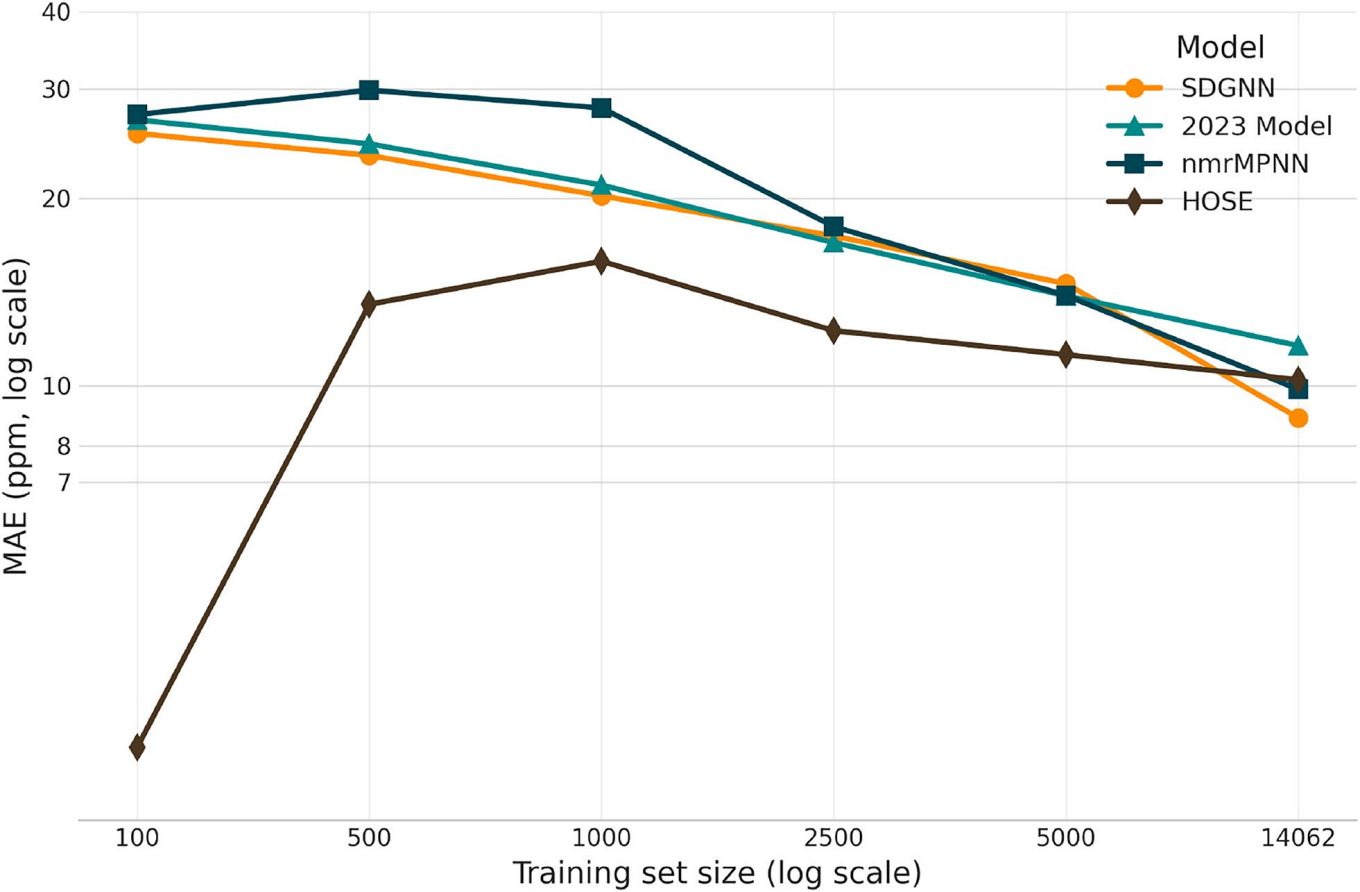
Visualization of model performance as a function of training set size using the results from Table 7. Mean absolute error (MAE) averaged across 10 folds. For HOSE, the reported MAE is conditional on successful fragment matches

Table 7 and Fig. 3 summarize prediction accuracy and model size across training set sizes ranging from 100 to 14,062 individual ^31^P shifts. The three GNNs SDGNN, 2023 Model and the nmrMPNN are compared against a lookup-based HOSE baseline.

In addition to reporting MAE and RMSE as mean ± standard deviation over 10 independent repeated SMILES grouped based splits, we analyze the distribution of absolute prediction errors by giving quantils in Table 9. The resulting variability across splits provides an empirical estimate of epistemic uncertainty due to data sampling and model initialization.

A clear trend across all three machine learning models is the consistent and significant improvement in predictive accuracy as the number of training samples increases. For instance, the SDGNN model’s MAE improves by almost a factor of three, decreasing from 25.45 ppm with 100 samples to a state-of-the-art 8.88 ppm on the full 14,062-sample dataset. This trend is accompanied by a marked reduction in the standard deviation of the error metrics, indicating that the models not only become more accurate but also more stable and reliable with access to more data. In contrast, HOSE codes show competitive MAE in the extreme low-data regime, but only modest improvement with more data accompanied with a very high failure rate of for example $80 \pm 10 \%$ for $k=100$. In scenarios with limited data (ranging from 100 to 500 samples), the machine learning models show a significantly larger standard deviations e.g. an MAE of 27.3 ± 12.5 ppm for the nmrMPNN on n=100 showing problems in generalizing from sparse-data regimes. All GNNs show a large enhancement in robustness with increased data size. Meanwhile, HOSE codes achieve the lowest MAE of $2.62 \ \!\pm \! \ 2.3$ ppm in the low-data scenario. However, a direkt comparison to the GNN models isn’t appropriate because of the enormous failure rate of HOSE codes. Even at 14,062 data points, the HOSE code based model exhibits a failure rate of $12.4 \pm 0.7 \%$ Consequently, the reported error metrics likely strongly overestimate the true performance of HOSE codes in all data scenarios, as molecules without valid predictions are excluded from the error calculation.

In the low data scenario from 100 to 1,000 samples, the SDGNN outperforms all other GNNs by improving its MAE from 25.45 to 20.2 ppm with relatively small parameter budgets of 0.10 and 0.01 M trainable parameters, indicating strong inductive bias and effective capacity control.

At full scale, $n=14,062$, the SDGNN outperforms all other GNNs as well as the HOSE code-based approach, achieving an MAE of 8.88 ± 0.4 while having the lowest parameter count of 0.36 M trainable parameters. Consequently, the SDGNN represents the best-performing model to date for the prediction of ^31^P chemical shifts.

To contextualize the performance of the SDGNN, we benchmarked it against established empirical and quantum-chemical approaches. As shown in Table 7 the SDGNN achieves an 10-fold average RMSE of 22.41 ppm on the test set on $k=14,062$, outperforming the HOSE code baseline which had a RMSE of 24.37 ppm (which is probably overly optimistic for HOSE since $\approx 12.4 \%$ of the molecules failed). Remarkably, the SDGNN also compares favorably to quantum mechanical methods used in high-throughput settings. Recent large-scale benchmarks on a subset of 10,007 molecule of the Ilm-NMR-P31 dataset, report an RMSE of 30.82 ppm for single-point DFT calculations in vacuum [40]. While the evaluation protocols differ, these results suggest that SDGNN achieves lower error than standard vacuum single-point DFT in this setting, at substantially lower computational cost. On a single CPU node, the SDGNN with the hyperparameters for $k=14,062$ achieves 258.9 molecules/s for model-only inference on precomputed graphs. End-to-end processing from SMILES reaches 16.7 molecules/s, where graph construction dominates $92.3\%$ of the runtime. Compared to the DFT-based workflow with 18.6 min per molecule on 16 CPU cores, the SDGNN provides a speedup of $\approx 1.9 \cdot 10^4$ for end-to-end to $\approx 2.9 \cdot 10^5$ for model-only.

**Table 8:** Comparison of SDGNN-derived substituent increments with literature values for representative phosphorus compound classes

Compound type	$B_{\text {lit}}$(ppm)	$B_{\text {GNN}}$ (ppm)	$A_{\beta ,\text {lit}}$ (ppm)	$A_{\beta ,\text {GNN}}$ (ppm)	$A_{\gamma ,\text {lit}}$ (ppm)	$A_{\gamma ,\text {GNN}}$ (ppm)	$R^2$
RPH$_2$	$-163.5$	$-163.2$	$+35.5$	$+31.1$	$-7.0$	$-7.0$	1.000
R$_2$PH	$-99.0$	$-99.4$	$+22.0$	$+22.5$	$-10.0$	$-7.6$	0.999
R$_3$P	$-62.0$	$-60.7$	$+13.5$	$+13.6$	$-4.0$	$-4.3$	0.989
R$_3$PH$^+$	$-3.2$	$-7.8$	$+8.6$	$+5.3$	$-3.0$	$-1.7$	0.892
R$_4$P$^+$	$+25.3$	4.6	$+3.7$	$+7.3$	$-1.5$	$-2.4$	0.997
R$_3$PO	$+36.2$	34.6	$+4.0$	$+5.5$	$-1.0$	$-0.7$	0.891

The parameters are obtained from linear fits. Literature values are taken from [38]. Charged species (R$_3$PH$^+$, R$_4$P$^+$) show larger deviations from literature constants

**Table 9:** Functional-group error analysis of SDGNN under the grouped (canonical SMILES) split

Category	Subclass	*n*	MAE (ppm)	RMSE (ppm)	$\mathbf {q_{50}}$ (ppm)	$\mathbf {q_{95}}$ (ppm)
P(V) species						
Phosphonates	R(OR)_2_P=O	2,333	1.70	4.05	0.91	5.27
Phosphine oxides	R_3_P=O	606	2.68	6.76	1.26	9.02
Phosphates	(OR)_3_P=O	304	2.79	20.67	0.84	5.31
P(III) species						
Phosphines	R_3_P (Tertiary)	786	3.90	10.12	1.47	14.69
Problem cases						
Charged species	Formal charge $\ne 0$	569	16.58	30.62	5.85	69.12
Silyl-substituted	P-Si bond	114	9.42	18.80	4.93	33.18
Reference	All neutral molecules	13,641	5.19	14.65	1.80	20.29

Performance is reported by chemical subclass; the largest errors occur for charged species and silyl-substituted compounds

Table 8 and 9 summarize the error distribution of the SDGNN across major phosphorus functional groups. A clear dependence on the local coordination environment is observed. Rigid P(V) species are predicted with substantially lower error than trivalent phosphines. In particular, phosphonates exhibit the smallest mean absolute error of 1.70 ppm and a median of $q_{50} = 0.91$ ppm, followed by phosphine oxides with 2.68 ppm and phosphates with 2.79 ppm, whereas tertiary phosphines P(III), R$_3$P, achieving 3.9 ppm, show increased variance. This trend is chemically intuitive, as P(V) compounds possess tetrahedral, conformationally rigid geometries, while P(III) phosphines are more sensitive to bond-angle variations, lone-pair inversion, and steric effects [42].

Consistent with this interpretation, the largest deviations arise for formally charged species with an MAE of 16.58 ppm and silyl-substituted phosphines with 9.42 ppm, where missing counterion information and strong inductive effects are not explicitly represented in the molecular graphs. Benzyl-substituted systems, by contrast, are predicted with a comparatively low error 3.0 ppm, indicating that aromatic ring-current effects are captured well by the SDGNN.

Beyond functional group analysis, we evaluated whether the SDGNN recovers classical substituent trends in $^{31}$P NMR spectroscopy. For homologous alkyl series, SDGNN predictions were fitted to the empirical increment model

$$\delta _{\textrm{pred}} = B + n_{\beta } A_{\beta } + n_{\gamma } A_{\gamma }$$

Across neutral phosphines and related P(V) species, the learned coefficients reproduce the established deshielding $\beta$-effect ($A_{\beta } > 0$) and shielding $\gamma$-effect ($A_{\gamma } < 0$) with near-perfect linearity ($R^2 = 0.989$). The magnitudes of the inferred increments closely match experimental reference values reported by Berger et al. [38], with representative ranges of $A_{\beta }\approx +13$ to $+31$ ppm and $A_{\gamma }\approx -4$ to $-7$ ppm for neutral phosphorus compounds. Deviations are primarily observed for charged species. While quaternary phosphonium salts R_4_P^+^ preserve strong linearity $R^2=0.997$, they exhibit a systematic offset in the base value *B*, which we attribute to missing counterion and solvent information as well as to averaging over heterogeneous experimental conditions in the training data. Protonated species R_3_PH^+^ show reduced linearity ($R^2=0.892$), consistent with the strong environment dependence of the P-H bond and enhanced sensitivity to local electrostatics. Taken together with the Bemis Murcko scaffold split performance of the SDGNN with an average MAE of 10.88 ppm (see supporting informations Table 7), these results suggest that the SDGNN does not merely interpolate, but internalizes chemically meaningful structure–property relationships.

**Fig. 4:**
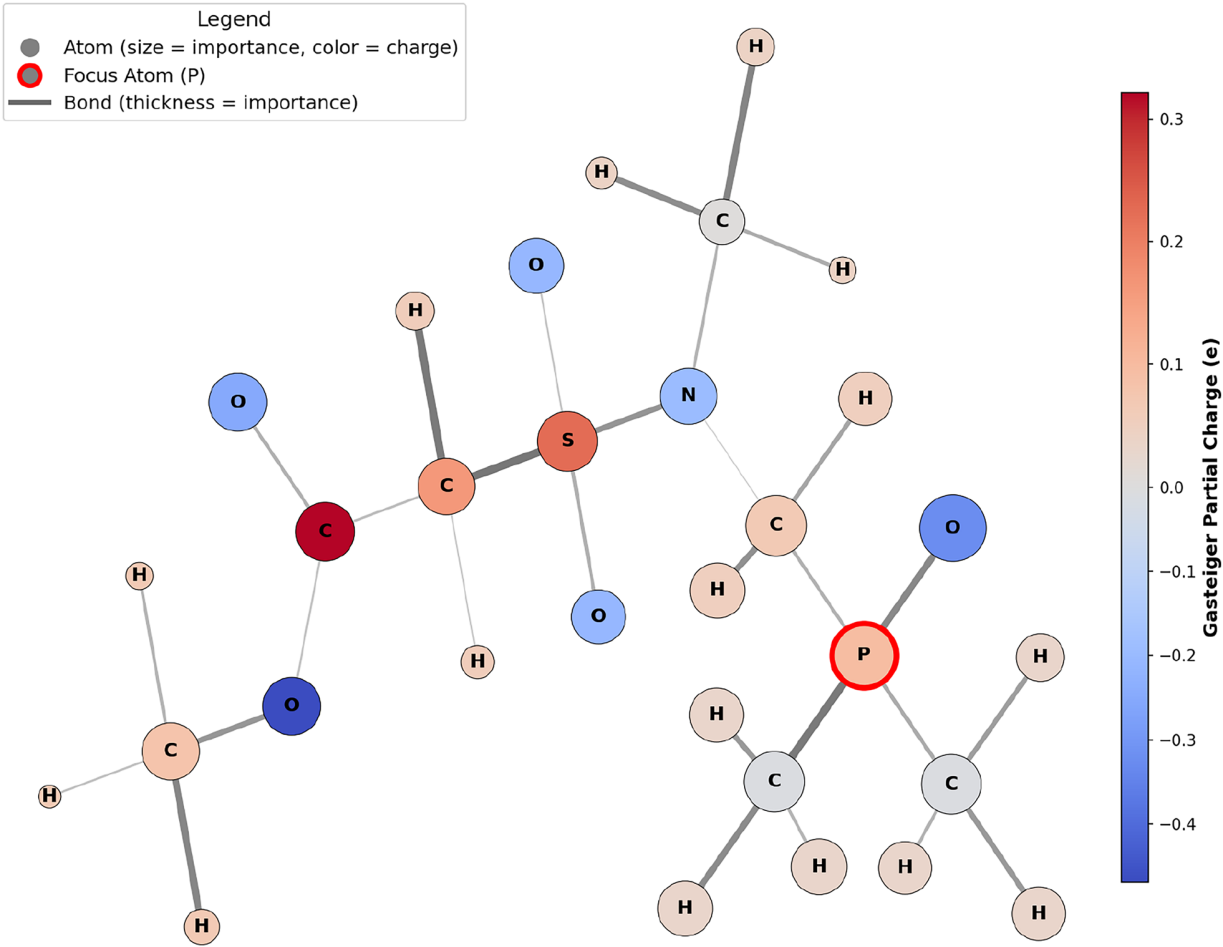
Visual explanation of one of the best nmrMPNN predictions with $\delta _{\text {pred}} = 43.8 \,\text {ppm}$ and $\delta _{\text {exp}} = 43.8 \,\text {ppm}$. As expected atoms that are further away from the phosphorous atom contribute less to the ^31^P chemical shift prediction

**Fig. 5:**
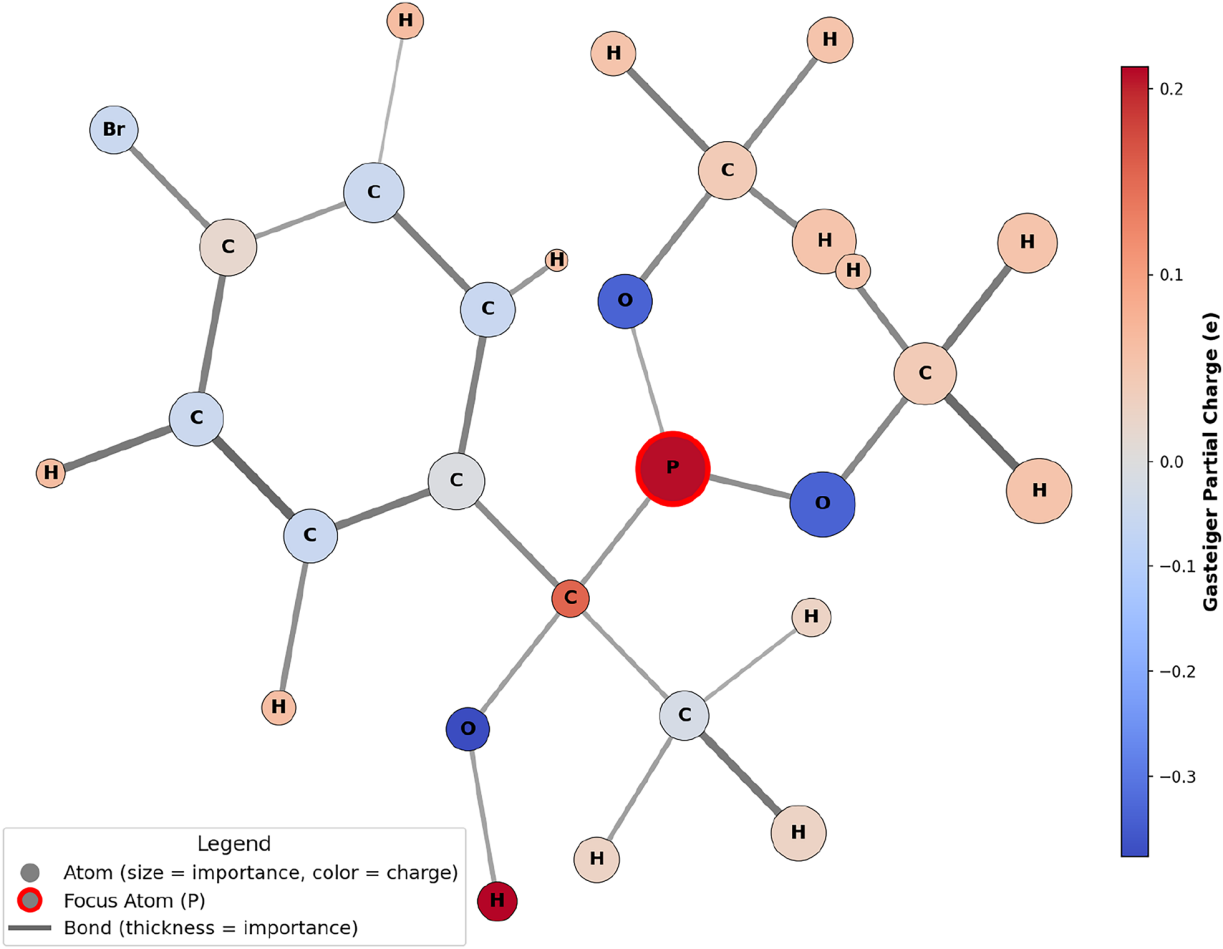
Visual explanation of one of the worst nmrMPNN predictions with $\delta _{\text {pred}} = 78.45 \,\text {ppm}$ and $\delta _{\text {exp}} = 406.20 \,\text {ppm}$. Surprisingly, the methyl carbon atoms are the most relevant nodes for the prediction

To gain molecular-level insight into the prediction results, atomic and bond importance was visualized using GNNExplainer [37], applied to the nmrMPNN trained on $k=10,000$ data points with a MAE of 9.54 ppm. Although the Gasteiger charges used as input features are an approximation, they provide a physically meaningful estimate of the electrostatic environment. In the following visualizations (Figs. 4 and 5), their color coding thus serves as a valuable aid to relate the importance of nodes and edges identified by the GNNExplainer to established chemical intuition. Additional GNNExplainer visualizations are provided in the Supporting Information.

Figure 4 depicts an asymmetric phosphine oxide. As expected, atoms farther from the phosphorous atom, such as the methyl ester group, are less important to the ^31^P chemical shift prediction. This behavior is analogous to HOSE codes, in which atoms closer to the atom of interest exert a stronger influence on the prediction than those farther away. Furthermore, Fig. 4 reveals that all atoms contribute almost evenly to the GNN prediction. The most important atom in Fig. 4 is the oxygen directly bound to the phosphorous, which aligns with the chemical intuition that the electronegative oxygen atom strongly affects the electron density at the phosphorous atom and thus the ^31^P shift. While the electron density at the phosphorus atom constitutes a relevant node feature for the prediction, no direct correlation can be established between an atom’s electron density and its importance in the GNN-based prediction.

Compared to the nodes for which a slight distance dependency can be observed, there is an almost random distribution of the bond importance beyond the first layer of bonds directly linked to the phosphorus atom. E.g. it is difficult to explain why the S-C bond is more important than the S-N although the latter is closer to the phosphorous atom. This emphasizes that although GNNs partly follow established physical concepts for the interpretation of chemical shifts, GNNs also learn non-linear patterns which are currently difficult to rationalize.

In contrast, Fig. 5 shows a case in which the ^31^P prediction is off by more than 100 ppm. The structure of the dimethyl phosphonite does not seem to be a particular difficult molecule from a human perception, yet the GNN has difficulties correctly predicting the ^31^P shift. The phosphorus atom is highly electron-deficient which can be explained by the high electronegativity of the neighbouring oxygen atoms. This might also explain why the directly bound carbon is also partially positively charged. Surprisingly, the GNNExplainer analysis indicates that the methyl groups, rather than the oxygen atoms, are identified as the most important nodes. As observed previously, the edge importance does not follow a clear pattern as the C-C bond between the ipso and ortho carbons is deemed most important. Overall, it remains unclear why the GNN fails so drastically in predicting the ^31^P shift for dimethyl phosphonite.

The application of GNNExplainer to the molecular graphs is an important step towards explainable artificial intelligence in the context of molecules. While the GNN presented here captures some molecular features that a chemist would also consider relevant, it occasionally highlights aspects that appear random or non-intuitive from a human perspective.

**Fig. 6:**
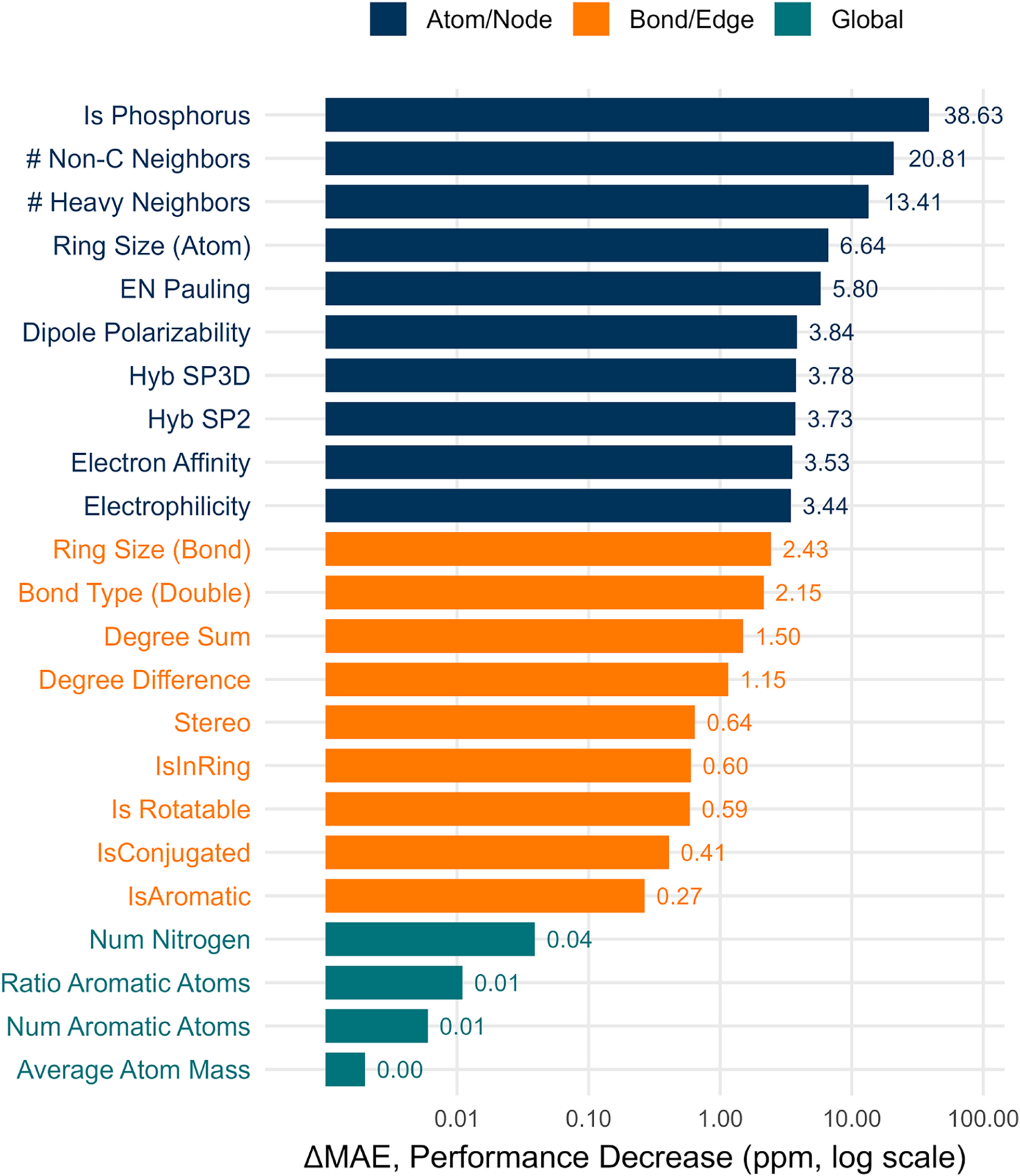
Most important node, edge, and global features for the SDGNN based on feature ablation. Node features dominate the performance of the SDGNN model. Edge features are less important than node features, but still contribute meaningfully when it comes to fine-tuning a ^31^P chemical shift prediction. Global features only play a minor role when predicting ^31^P chemical shifts

The feature ablation study confirms that the the SDGNN’s predictions are governed by chemically intuitive, local structural features. As shown in Fig. 6, the identity of the central atom has the largest influence, with the presence of phosphorus itself leading to an increase in mean absolute error of 38.6 ppm upon removal. The number of non-carbon neighbors and the number of heavy (non-hydrogen) neighbors surrounding the phosphorus atom are also highly influential, contributing 20.8 ppm and 13.4 ppm, respectively. The local ring environment, reflected by the atomic ring size, further affects the predicted chemical shift by 6.6 ppm. Electronegativity, dipole polarizability and hybridization state are also important but to a lesser extent. Finally, electron affinity and electrophilicity provide additional, though smaller, contributions to the prediction of the ^31^P chemical shift. As shown in Fig. 6, the edge features are overall much less important than the node features. The most critical information is derived from the topology of substituents, particularly the presence and size of ring systems with an MAE decrease of 2.4 ppm. Beyond that the immediate coordination environment of the phosphorus atom plays an important role: Removing the aggregate bond order and the sum of atomic degrees produces the second-largest performance degradation, with a $\Delta$MAE values of 2.2 ppm and 1.5 ppm, respectively. This highlights the model’s reliance on molecular connectivity to infer the electronic state of the phosphorus center. In contrast, global molecular properties that are not directly related to the local phosphorus environment, such as the total nitrogen count or the ratio of aromatic atoms, have a negligible impact (Fig. 6). These results strongly support that the model correctly learns that the ^31^P chemical shift is predominantly governed by local electronic effects. The model’s emphasis on coordination and bonding patterns reflects established chemical principles, wherein the phosphorus oxidation state and the $\sigma /\pi$ character of P-X bonds dominate nuclear shielding [36]. Similarly, the importance of ring descriptors effectively captures known electronic perturbations from aromatic and heterocyclic groups, such as ring-current effects.

**Fig. 7:**
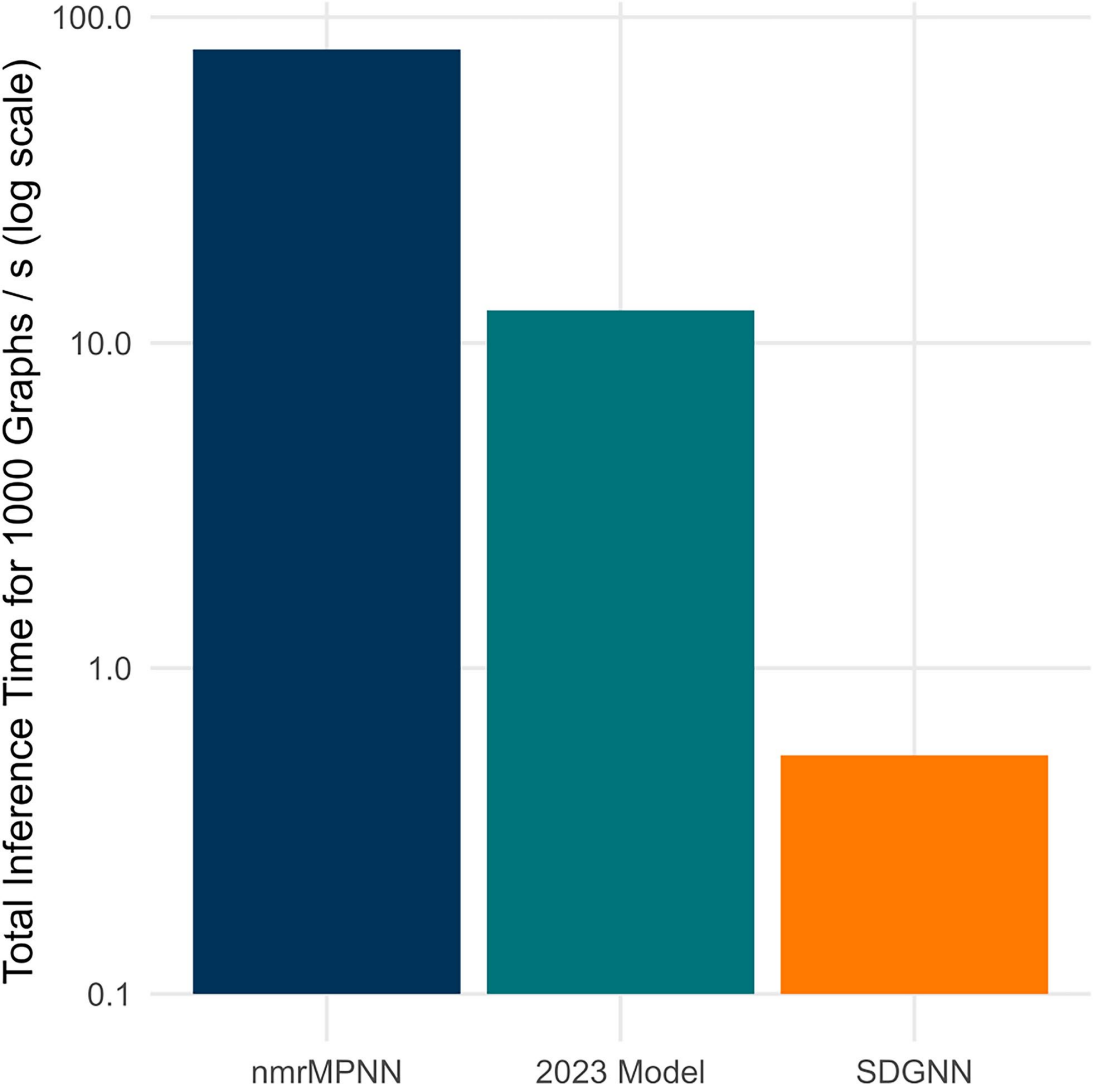
The SDGNN model demonstrates vastly superior inference speed, capable of processing more than 1,700 molecules per second. It is over ten times faster than the 2023 model and more than 120 times faster than the nmrMPNN on the preprocessed Ilm-31P dataset

Beyond predictive accuracy, inference speed dictates a model’s utility in real-world workflows. The SDGNN model’s efficiency (Fig. 7) elevates it from a simple validation tool to a powerful discovery engine. This advantage becomes decisive at scale: screening a one-million-compound library requires only 10 min with the SDGNN, compared to 1.7 h for the next-fastest model. This acceleration makes it feasible to perform high-throughput screening of billion-compound virtual libraries, annotate entire chemical databases, and integrate the model into interactive design tools, fundamentally enabling large-scale chemical discovery.

## Conclusion

This study investigated graph-based prediction of ^31^P chemical shifts on the Ilm-NMR-P31 dataset. A new Small-Data GNN (SDGNN) based on a MetaLayer architecture was introduced. Compared to existing small-data and general GNNs for the prediction of NMR shifts, the newly introduced SDGNN achieved the highest predictive performance for ^31^P. The SDGNN is particularly well-suited for small datasets because it enforces a strict locality constraint by enforcing a single message-passing round and avoids parameter inflation through a compact update mechanism, unlike benchmark models that employ feature concatenation or deeper architectures which are prone to overfitting in low-data regimes. We demonstrated that this approach not only outperforms empirical HOSE code baselines but also surpasses standard vacuum density functional theory (DFT) calculations in both accuracy and inference speed.

We compared accuracy, scaling with dataset size, parameter efficiency, inference throughput, as well as the explainability of the prediction results. Feature importance analyses, including GNNExplainer and ablation studies, further confirmed that the SDGNN learns chemically intuitive principles, focusing on the local electronic and structural environment of the phosphorus atom to make its predictions. Importantly, chemical consistency analyses revealed that the model implicitly rediscovers classical substituent trends, including the deshielding $\beta$-effect and shielding $\gamma$-effect, with learned increments matching experimental reference ranges. Functional-group–resolved error analysis further showed that rigid P(V) species such as phosphonates and phosphine oxides are predicted with particularly high accuracy, whereas larger deviations occur for charged compounds and sterically or electronically complex motifs.

Despite these strong results, we acknowledge several limitations. The labels originate from heterogeneous experimental conditions and likely contain noise. Our global features only coarsely capture such context. Because the models operate on 2D graphs and use approximate charges, through-space and conformational effects are only indirectly represented. As a consequence, prediction errors increase for molecules with unusual coordination environments or extreme chemical shifts. Finally, the current study focuses exclusively on ^31^P NMR, and generalization to other nuclei remains to be explored.

Future research will proceed along several promising directions. To improve robustness and coverage of chemical space, we plan to integrate active learning strategies that target underrepresented or high-uncertainty regions. A key objective is to extend the SDGNN framework to additional NMR observables, including J-couplings, as well as to other NMR-active nuclei such as ^11^B and ^103^Rh. Finally, the model’s high inference speed enables deployment as an open-access interactive web platform, providing the community with real-time spectral predictions and supporting data-driven molecular design.

## Supplementary Information

Supplementary material 1.

Supplementary material 2.

Supplementary material 3.

Supplementary material 4.

Supplementary material 5.

Supplementary material 6.

Supplementary material 7.

Supplementary material 8.

## Data Availability

The Ilm-NMR-P31 dataset is available online at the GitHub repository https://github.com/clacor/Ilm-NMR-P31. The dataset is also available from Zenodo https://doi.org/10.5281/zenodo.8260783. The Python files used for the model analysis are available as Additional Files.
