# Supplementary material for: A light-weight Graph Neural Network for the prediction of 31P Nuclear Magnetic Resonance signals
Source: J Cheminform. 2026 Mar 17;18:49. doi: 10.1186/s13321-026-01178-6 (PMC13112750; doi:10.1186/s13321-026-01178-6)
Supplement: Supplementary file 4 — Supplementary material 4. [file 13321_2026_1178_MOESM4_ESM.pdf]

# Supporting Information: A Light-Weight Graph Neural Network for the Prediction of $^{31}$P Nuclear Magnetic Resonance Signals

Dimitri Domnjuk[1,2], Jana de Wiljes[2,3*], Robert Geitner[1*]

[1]Institute of Chemistry and Bioengineering, Group of Physical Chemistry/ Catalysis, Technische Universität Ilmenau, Weimarer Str. 32, Ilmenau, 98693, Germany.

[2]Institute of Mathematics, Group of Mathematics in Data Science, Technische Universität Ilmenau, Weimarer Str. 25, Ilmenau, 98693, Germany.

[3]School of Engineering Sciences, Department of Computational Engineering, LUT University, Yliopistonkatu 34, Lappeenranta, 53850, Finland.

*Corresponding author(s). E-mail(s): jana.de-wiljes@tu-ilmenau.de; robert.geitner@tu-ilmenau.de;
Contributing authors: dimitri.domnjuk@tu-ilmenau.de;

# 1 Additional Data and Visualizations

The visualizations of the explanations consistently assign a high importance to the P atom and its first coordination sphere, P-X bonds, which is in line with $^{31}$P chemical shifts being dominated by the local electronic environment.

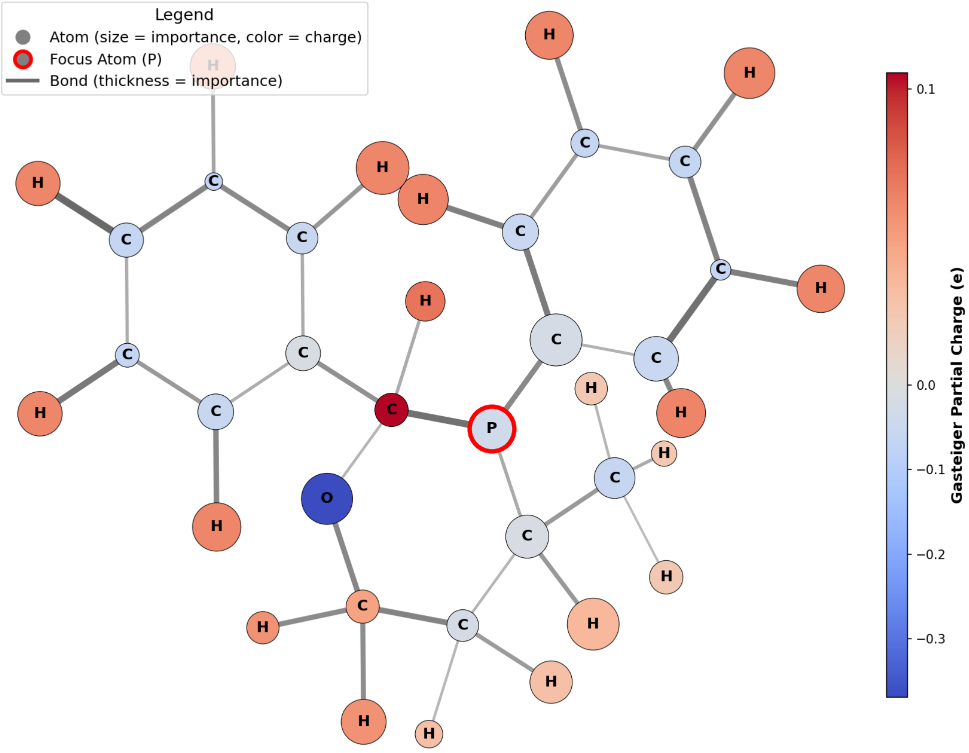


**Fig. S1** Visual explanation of one of the best nmrMPNN predictions with $\delta_{\mathrm{pred}} = -11.8\,\mathrm{ppm}$ and $\delta_{\mathrm{exp}} = -11.8\,\mathrm{ppm}$.

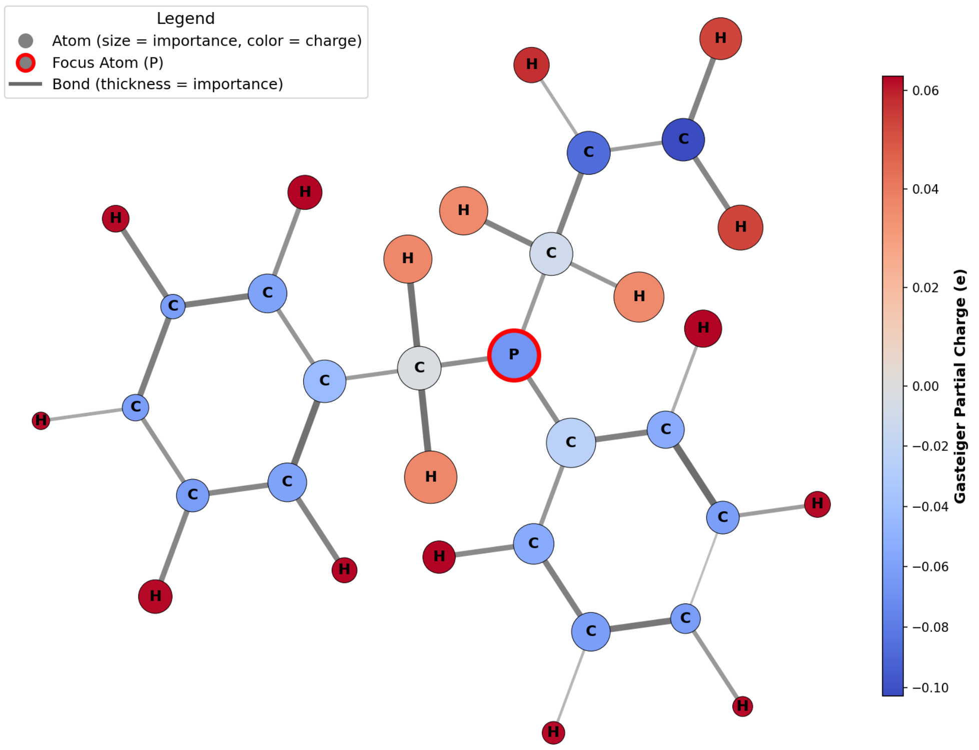


**Fig. S2** Visual explanation of one of the best nmrMPNN predictions with $\delta_{\text{pred}} = -20.8$ ppm and $\delta_{\text{exp}} = -20.7$ ppm.

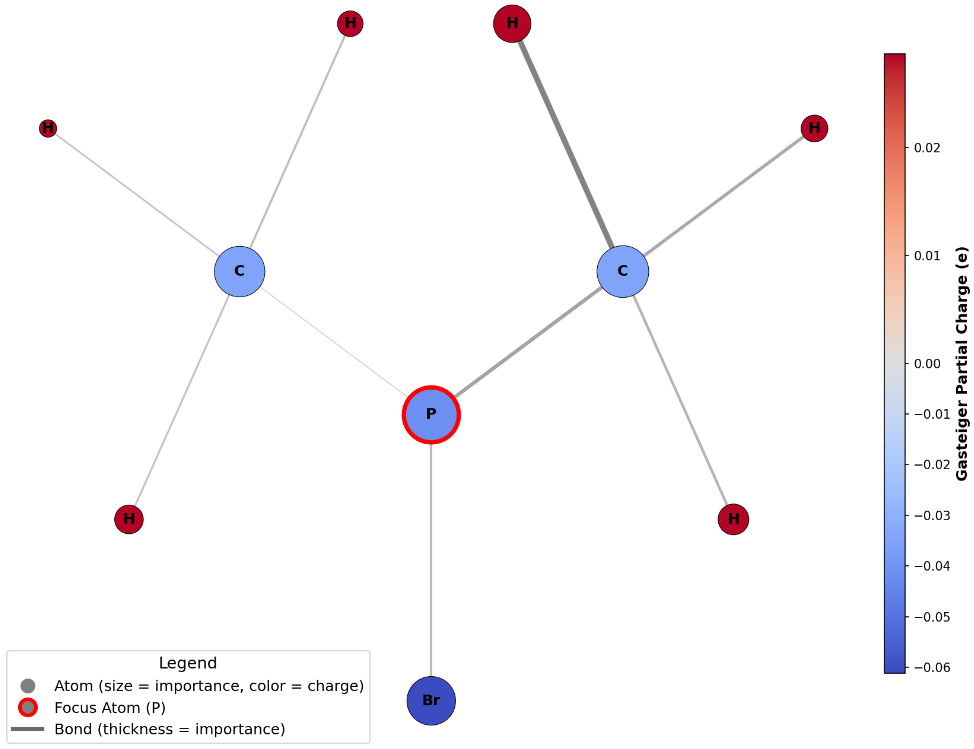


**Fig. S3** Visual explanation of one of the worst nmrMPNN predictions with $\delta_{\mathrm{pred}} = 84.7\,\mathrm{ppm}$ and $\delta_{\mathrm{exp}} = -37.9\,\mathrm{ppm}$.

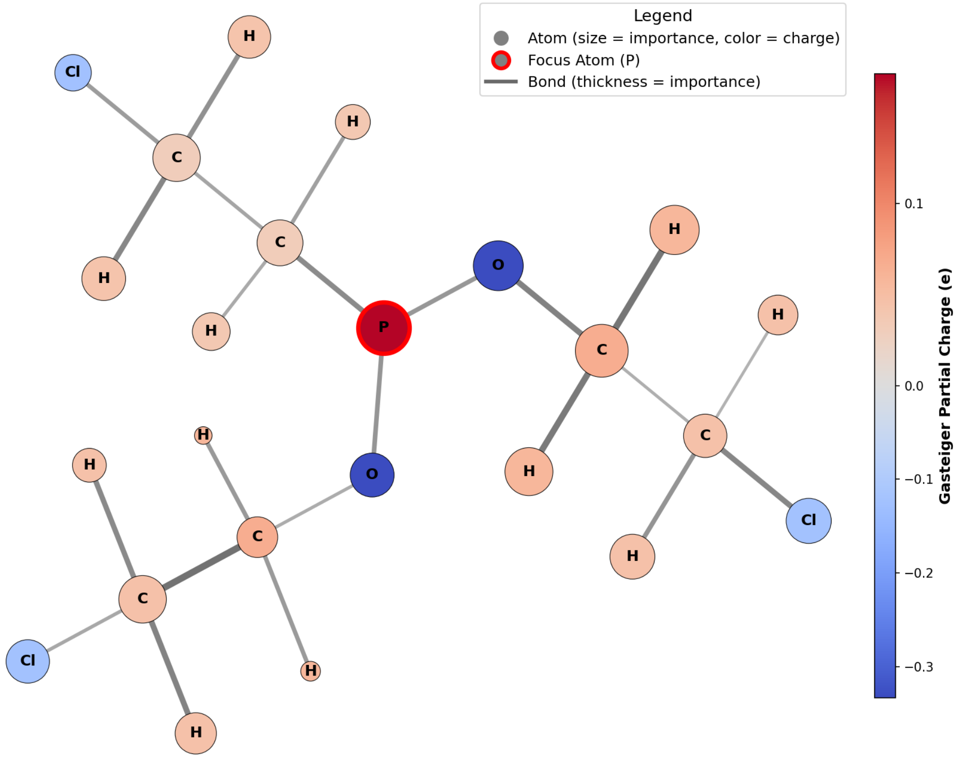


**Fig. S4** Visual explanation of one of the worst nmrMPNN predictions with $\delta_{\text{pred}} = 144.9\,\text{ppm}$ and $\delta_{\text{exp}} = 26.4\,\text{ppm}$.

**Table S1** Scaffold Split Results. Model performance evaluated using a Bemis-Murcko scaffold split. This chemically informed splitting tests the generalization to entirely new molecular skeletons not seen during training. As expected, errors are higher compared to the grouped split due to the lack of global structural overlap.

| **Model** | **Metric** | **100** | **500** | **1000** | **2500** | **5000** | **14062** |
|---|---|---|---|---|---|---|---|
| **SDGNN** | MAE (ppm) | 26.84 ± 8.1 | 25.83 ± 8.1 | 20.18 ± 6.0 | 18.04 ± 4.29 | 14.91 ± 1.9 | 10.88 ± 2.9 |
| | RMSE (ppm) | 37.78 ± 12.0 | 47.93 ± 17.2 | 33.84 ± 14.6 | 36.81 ± 10.83 | 29.88 ± 6.8 | 23.55 ± 6.3 |
| **2023 Model** | MAE (ppm) | 25.33 ± 11.6 | 26.08 ± 8.8 | 20.93 ± 6.4 | 18.09 ± 4.36 | 15.27 ± 2.4 | 13.50 ± 2.7 |
| | RMSE (ppm) | 35.59 ± 17.7 | 47.46 ± 20.1 | 36.91 ± 14.1 | 33.85 ± 9.95 | 29.67 ± 7.1 | 28.42 ± 6.1 |
| **nmrMPNN** | MAE (ppm) | 25.70 ± 5.0 | 30.69 ± 7.3 | 26.19 ± 7.8 | 19.62 ± 4.78 | 14.90 ± 2.2 | 12.03 ± 2.6 |
| | RMSE (ppm) | 37.72 ± 11.2 | 53.83 ± 17.1 | 44.04 ± 15.9 | 35.43 ± 9.41 | 29.74 ± 5.7 | 25.15 ± 6.0 |
| **HOSE** | MAE (ppm) | 4.31 ± 4.9 | 16.28 ± 9.7 | 14.37 ± 5.4 | 12.36 ± 1.7 | 13.21 ± 1.8 | 12.74 ± 0.9 |
| | RMSE (ppm) | 4.91 ± 5.4 | 29.76 ± 20.3 | 24.99 ± 13.7 | 24.28 ± 5.0 | 28.02 ± 5.6 | 27.26 ± 3.6 |
| | Failed (%) | 80.6 ± 9.6 | 66.8 ± 8.5 | 55.1 ± 8.7 | 45.4 ± 7.8 | 35.2 ± 7.2 | 23.2 ± 3.9 |

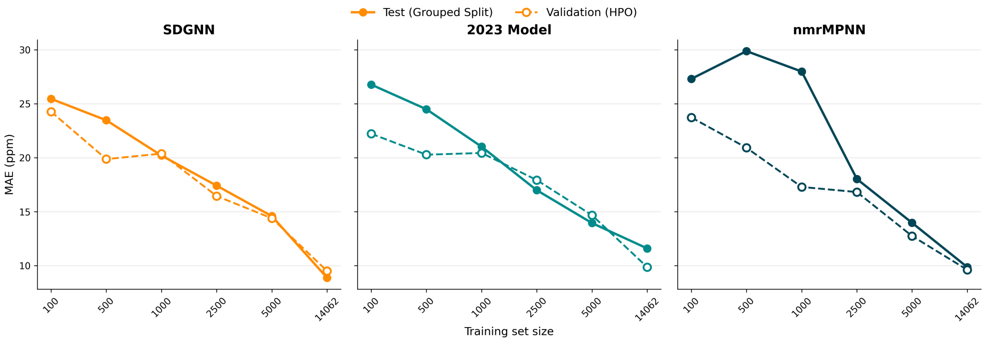


**Fig. S5** Validation vs. Test MAE trajectories. Comparison of model performance across training set sizes. Dashed lines represent the average validation loss from the grid search 3-fold cross validation, while solid lines depict the performance on the independent, grouped 10-fold cross validation test set. The convergence of curves at N = 14,062 confirms that the models successfully learn transferable chemical representations when provided with sufficient training data.

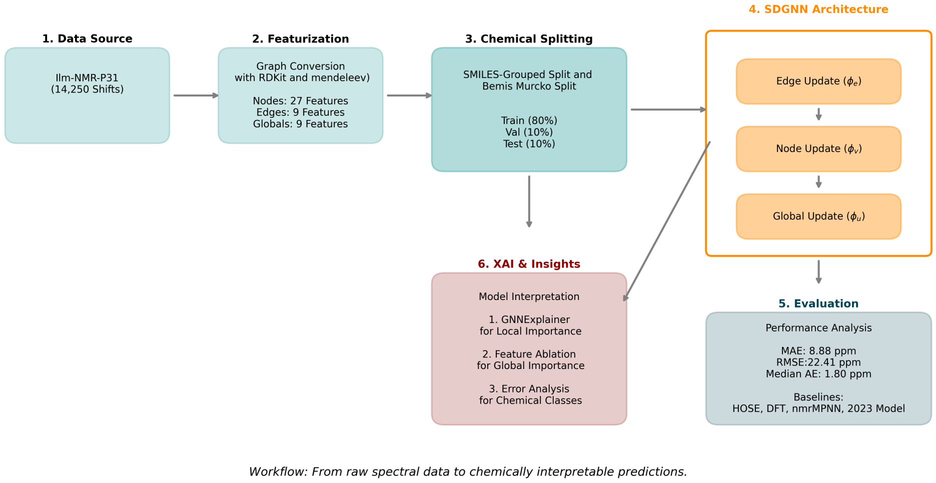


**Fig. S6** Schematic overview of the proposed computational workflow: The pipeline begins with the (1) curation of the Ilm-NMR-P31 dataset, followed by (2) building the graph representation and features using atom, bond, and global descriptors. (3) Chemical splitting, grouped by SMILES and a stricter Bemis Murko split is applied to prevent data leakage. The core model is the (4) Small Data GNN (SDGNN), employing a parameter-efficient MetaLayer architecture. Finally, the model is subjected to (5) extensive benchmarking against empirical (HOSE) and quantum-chemical (DFT) baselines, and analyzed via (6) Explainable AI (XAI) techniques including GNNExplainer and functional group error analysis.

# 2 Model Definitions

## 2.1 2023 Model

We implement a MetaLayer framework established GNN for the sparse data scenario based on [1] with explicit edge, node, and global blocks. A molecule is represented as a graph $\mathcal{G} = (V, E, X, E_{\text{feat}})$ with node features $x_v \in \mathbb{R}^{d_x}$ and edge features $e_{uv} \in \mathbb{R}^{d_e}$. In our implementation, the global state is initialized as a learnable latent vector $u^{(0)} \in \mathbb{R}^1$ set to zero per graph.
Node and edge features are mapped to latent dimensions via MLP encoders

$$h_v^{(0)} = \text{Enc}_x(x_v) \in \mathbb{R}^{d_n}, \qquad a_{uv}^{(0)} = \text{Enc}_e(e_{uv}) \in \mathbb{R}^{d'_e},$$

where $d_n$ and $d'_e$ are implementation-specific hidden sizes (in code: $d_n = \lfloor d/2 \rfloor$, $d'_e = \lfloor d/4 \rfloor$). The initial global state is

$$u^{(0)} = \mathbf{0} \in \mathbb{R}^1.$$

We perform $L$ rounds of block updates. In round $l = 1, \dots, L$, the edge block updates edge latents by an MLP:

$$a_{uv}^{(l)} = \phi_e\Big(\big[h_u^{(l-1)} \parallel h_v^{(l-1)} \parallel a_{uv}^{(l-1)}\big]\Big) \in \mathbb{R}^{d'_e}.$$

The node block aggregates incoming edge latents by a sum at the destination node and applies an MLP:

$$m_v^{(l)} = \sum_{u \in \mathcal{N}(v)} a_{uv}^{(l)}, \qquad h_v^{(l)} = \phi_n\Big(\big[h_v^{(l-1)} \parallel m_v^{(l)}\big]\Big) \in \mathbb{R}^{d_n}.$$

The global block pools node latents per graph by sum and updates the global state:

$$s^{(l)} = \sum_{v \in V} h_v^{(l)}, \qquad u^{(l)} = \phi_g\Big(\big[u^{(l-1)} \parallel s^{(l)}\big]\Big) \in \mathbb{R}^{d_g}.$$

Notably, this baseline global update depends only on the pooled node state (no explicit edge pooling term is used).
After $L$ rounds, a graph-level MLP maps the final global state to the scalar prediction:

$$\hat{y} = \psi\Big(u^{(L)}\Big), \qquad \psi : \mathbb{R}^{d_g} \to \mathbb{R}.$$

## 2.2 SDGNN

We employ a single-pass meta-layer GNN that updates edge, node, and global representations within one block. A molecule is represented as a graph $\mathcal{G} = (V, E, X, E_{\text{feat}}, u)$ with node features $x_v \in \mathbb{R}^{d_x}$, edge features $e_{uv} \in \mathbb{R}^{d_e}$, and a per-graph feature vector

$u \in \mathbb{R}^{d_u}$. The model uses a hidden dimension $d$ for intermediate edge and global representations.
For each directed edge $(u \to v)$, an edge MLP produces an edge embedding:

$$\tilde{e}_{uv} = \phi_e\big([x_u \,\|\, x_v \,\|\, e_{uv}]\big) \in \mathbb{R}^d,$$

where $\phi_e : \mathbb{R}^{2d_x+d_e} \to \mathbb{R}^d$.
Incoming edge embeddings are aggregated by sum at each destination node and concatenated with the original node features:

$$m_v = \sum_{u \in \mathcal{N}(v)} \tilde{e}_{uv} \in \mathbb{R}^d, \qquad \tilde{x}_v = \phi_n\big([x_v \,\|\, m_v]\big) \in \mathbb{R}^d,$$

with $\phi_n : \mathbb{R}^{d_x+d} \to \mathbb{R}^d$. Thus, SDGNN maps the input node features $x_v$ to an updated node embedding $\tilde{x}_v$ in $\mathbb{R}^d$.
Node embeddings are pooled per graph by sum and combined with the graph-level descriptors:

$$s = \sum_{v \in V} \tilde{x}_v \in \mathbb{R}^d, \qquad \tilde{u} = \phi_g\big([u \,\|\, s]\big) \in \mathbb{R}^d,$$

where $\phi_g : \mathbb{R}^{d_u+d} \to \mathbb{R}^d$.
A final linear layer maps the global embedding to the scalar prediction:

$$\hat{y} = w^\top \tilde{u} + b.$$

## 2.3 nmrMPNN

For comparison, we adapt a conventional message passing GNN to graph-level prediction following [2]. A molecule is represented as a graph $\mathcal{G} = (V, E, X, E_{\text{feat}}, u)$ with node features $x_v \in \mathbb{R}^{d_x}$, edge features $e_{uv} \in \mathbb{R}^{d_e}$, and a per-graph feature vector $u \in \mathbb{R}^{d_u}$.
Initial node states are obtained via a learnable linear projection

$$h_v^{(0)} = W_p\, x_v \in \mathbb{R}^d,$$

where $d$ denotes the hidden dimension.
We perform $T$ message-passing rounds. At step $t = 1, \ldots, T$, messages to $v$ are computed by an edge network $\Theta : \mathbb{R}^{d_e} \to \mathbb{R}^{d \times d}$ and additive aggregation:

$$m_v^{(t)} = \sum_{u \in \mathcal{N}(v)} \Theta(e_{uv})\, h_u^{(t-1)}.$$

Node states are updated recurrently via a GRU update. In the implementation this is realized with a GRU module applied at sequence length 1, which is equivalent to a GRUCell update at each step:

$$h_v^{(t)} = \mathrm{GRU}\big(m_v^{(t)},\, h_v^{(t-1)}\big).$$

To capture information at multiple message-passing radii, we concatenate all intermediate states $\tilde{h}_v = \big[h_v^{(0)}; \ldots; h_v^{(T)}\big] \in \mathbb{R}^{d(T+1)}$ and aggregate them with a Set2Set readout $S(\cdot)$:

$$s = S\big(\{\tilde{h}_v\}_{v \in V}\big) \in \mathbb{R}^{2d(T+1)}.$$

The graph embedding is concatenated with the global features,

$$g = \big[s; u\big] \in \mathbb{R}^{2d(T+1)+d_u},$$

and passed to an MLP head to produce the scalar prediction $\hat{y} = f(g)$.

# References


[1] Rull, H., Fischer, M., Kuhn, S.: Nmr shift prediction from small data quantities. Journal of Cheminformatics **15**, 114 (2023) https://doi.org/10.1186/s13321-023-00785-x

[2] Kwon, Y., Lee, D., Choi, Y.-S., Kang, M., Kang, S.: Neural message passing for nmr chemical shift prediction. Journal of Chemical Information and Modeling **60**(4) (2020) https://doi.org/10.1021/acs.jcim.0c00195
